# Influences of comorbidities on perioperative rehabilitation in patients with gastrointestinal cancers: a retrospective study

**DOI:** 10.1186/s12957-023-03207-2

**Published:** 2023-10-26

**Authors:** Naoto Seriu, Shinji Tsukamoto, Yukako Ishida, Nobuki Yamanaka, Tomoo Mano, Yasuyo Kobayashi, Marina Sajiki-Ito, Yusuke Inagaki, Yuu Tanaka, Masayuki Sho, Akira Kido

**Affiliations:** 1 Department of Rehabilitation Medicine, Nara Medical University, 840 Shijo-Cho, Kashihara, Nara 634-8522 Japan; 2 Department of Rehabilitation, Faculty of Health Science, Wakayama Professional University of Rehabilitation, Wakayama, Japan; 3 Department of Surgery, Nara Medical University, Nara, Japan

**Keywords:** Charlson Comorbidity Index, Rehabilitation therapy, Gastrointestinal cancers

## Abstract

**Background:**

Older patients are more likely to have comorbidities than younger patients, and multiple comorbidities are associated with mortality in patients with cancer. Therefore, we hypothesized that a functional comorbidity index could predict the therapeutic effects of rehabilitation.

**Objectives:**

In this study, we investigate whether the comorbidities influenced the execution and therapeutic effects of rehabilitation.

**Methods:**

A consecutive cohort of 48 patients with gastrointestinal cancer who underwent surgery between January 1 and November 30, 2020, was analyzed. Charlson Comorbidity Index (CCI) scores were calculated based on data derived from medical records. The primary outcomes were ambulation status, duration (days) from the start of postoperative rehabilitation, and length of hospital stay. We investigated the relationship between CCI scores and primary outcomes.

**Results:**

The CCI did not correlate with the duration of rehabilitation or the length of hospital stay. Subsequently, patients with functional recovery problems were evaluated, and we identified the conditions that were not included in the list using CCI scores. Most conditions are associated with surgical complications. Furthermore, using the Clavien-Dindo classification (CDC), we assessed the clinical features of the severity of complications. We found that the length of stay and the duration to start rehabilitation were significantly longer in the patients with higher severity of surgical complications (CDC≧III) than in those with lower severity (CDC≦II).

**Conclusions:**

Treatment-related conditions may significantly impact the perioperative period more than the original comorbidities. In addition to original comorbidities, events related to surgical complications should be assessed to determine the therapeutic effects of rehabilitation in patients with gastrointestinal cancer.

**Supplementary Information:**

The online version contains supplementary material available at 10.1186/s12957-023-03207-2.

## Introduction

The incidence of cancer in advanced countries has increased [1]. Surgery is the only curative option for patients with resectable cancers. Advances in less invasive surgery, such as the endoscopic approach, provide therapeutic opportunities, even for older patients [2]. However, older patients were more likely to have comorbidities than younger patients [3, 4]. In addition, diminished physiological reserves may cause complications after surgery [3, 4].

Perioperative rehabilitation has been promoted to prevent complications after surgery [5, 6]. Perioperative rehabilitation was reported to decrease the risks for respiratory complications [5, 6]. It facilitates ambulation and enables the earlier initiation of adjuvant therapy after surgery [5, 6]. Although perioperative rehabilitation is essential in older patients, comorbidities can interfere with physical training. For example, myocardial infarctions or congestive heart failure may affect the execution of endurance exercises. Connective disease or hemiplegia may affect the execution of muscle-strengthening exercises.

However, the effect of comorbidities on rehabilitation remains controversial. Several researchers have reported that comorbidities can predict poor functional recovery or prolonged hospital stay in patients with stroke [7, 8], hip fracture [8, 9], or coronary bypass graft [10]. In contrast, some studies have reported that comorbidities do not predict functional recovery after rehabilitation in patients with burn injuries [11] and spinal cord injuries [12], or in a mixed population with neurological conditions, deconditioning after acute events, and orthopedic conditions [13].

Multiple comorbidities affect the nutritional status after esophageal cancer surgery [14], induce infectious complications after gastric cancer surgery [15], and cause early mortality after pancreatic cancer surgery [16]. However, a previous study reported opposite findings in patients with bladder cancer [17]. There are few systematic reviews and meta-analyses on this topic. In the entity, no reports specify the predictive value of the comorbidity on the therapeutic effect of rehabilitation in patients with gastrointestinal cancer. Our study investigating the impact of comorbidity on cancer rehabilitation is based on this.

Recently, Chan et al. [18] investigated the association between comorbidity measures and mortality in geriatric rehabilitation inpatients according to the cancer status (no cancer, history of cancer, or active cancer). They investigated 693 patients and concluded that comorbidity measures were associated with a higher mortality risk using the Charlson Comorbidity Index (CCI).

In this study, we hypothesized that the CCI scores would predict the negative therapeutic effects of perioperative rehabilitation in patients with cancer. Higher scores would correlate with lower therapeutic effects because comorbidities affect training execution or training efficacy. Understanding the potential factors influencing rehabilitation is helpful for successful risk management during training. We retrospectively evaluated 48 patients with gastrointestinal cancer who underwent perioperative rehabilitation. We assessed whether the comorbidities influenced the execution and therapeutic effects of rehabilitation.

## Methods

### Study design

We retrospectively reviewed data from 48 consecutive patients with gastrointestinal cancer who underwent curative or palliative surgery and perioperative rehabilitation. We examined the type of cancer, CCI score, ambulation status, duration (in days) to start rehabilitation after surgery, and length of hospital stay.

### Participants

This retrospective study was conducted at the Nara Medical University Hospital. The study protocol was approved by the hospital’s Institutional Review Board (approval no. 3413). The study was conducted in accordance with the principles of the Declaration of Helsinki and the laws and regulations of Japan. Between January 1 and November 30, 2020, 98 patients with gastrointestinal cancer were referred to the department of rehabilitation medicine in our hospital. Of the patients, 50 were excluded from the study because they did not undergo the planned surgery but underwent exploratory laparotomy, endoscopic drainage, medication, or radiation for local control due to unexpected tumor progression. Finally, we analyzed 48 consecutive patients. All patients received a comprehensive palliative intervention from the beginning of treatment [19, 20]. The team of the identical surgical department performed all the procedures.

### Charlson Comorbidity Index scores

CCI scores were calculated according to the patients’ preoperative conditions derived from medical records [21]. Table 1 lists the weights of the CCI conditions. The sum of all the weights resulted in a single patient comorbidity score.

**Table 1:** Charlson comorbidity index [19]

Weight	Conditions
1	Myocardial infarction Congestive heart failure Peripheral vascular disease Cerebrovascular disease Dementia Chronic obstructive disease Connective tissue disease Ulcer disease Mild liver disease Diabetes mellitus
2	Hemiplegia Moderate/severe renal disease Diabetes with end-stage organ damage Any tumors without metastasis Leukemia Lymphoma
3	Moderate/severe liver disease
6	Metastatic solid tumor AIDS

### Clavien-Dindo classification

The Clavien-Dindo classification (CDC) ranks the severity of surgical complication [22–24]. It is based on the type of therapy required to correct complications. The scale comprises several grades. Grade I complications are usually mild; however, Grade II or higher complications are more severe. In this study, we defined complications of higher severity as those equal to or greater than Grade III and those of lower severity as those equal to or less than Grade II.

### Outcome evaluations

All the patients were admitted for surgery. The primary outcome evaluations were ambulation status, duration (days) to the start of rehabilitation post-surgery, and length of hospital stay. The level of mobility achieved during rehabilitation was defined as levels 1–5 as described by Kim et al. [25] with some modifications by Ishida et al. [26] (Table 2).

**Table 2:** Maximum levels of mobility during perioperative rehabilitation as described by Kim et al. [23]

Level 1	Therapeutic (in-bed) exercises
Level 2	Bed mobility (supine to sit)
Level 3	Transfer training (sit to stand/bed to chair)
Level 4	Gait training (walk with assistance)
Level 5	Gait training (walk independently)

### Rehabilitation

Rehabilitation was initiated after patients underwent medical examinations performed by a physiatrist. All patients are usually referred to the rehabilitation department the day after surgery or after the attending physician has confirmed that vital signs are stable. The physiatrist will assess the patient on the day of referral. The physiatrists evaluated the general condition of patients based on Gerber’s recommendation [27]. The ambulation exercises prescribed by the physiatrist included in-bed exercises, sitting, standing, and walking, with or without support. The weaning exercises were performed according to the patient’s general condition, respiration, and circulation. Once the patient could walk on the ward, muscle-strengthening exercises such as squats and heel raises were performed. In addition, those who were more active additionally performed bicycle ergometers as aerobic exercise. There were no adverse events in all patients during the rehabilitation treatment. The exercise lasted 20–40-min per session and was performed 5–6 times per week.

### Statistical analysis

Statistical analyses were performed using SPSS software (version 22.0; IBM Corp., Armonk, NY, USA). Statistical significance was set at *P* < 0.05. Spearman’s correlation test was used to evaluate the association between the CCI scores; duration of rehabilitation after surgery; and length of hospital stay; and between the age, duration of rehabilitation after surgery, and length of hospital stay. Mann–Whitney’s *U* test was used to evaluate the association between the severity of surgical complications and the CCI scores, duration of rehabilitation after surgery, and length of hospital stay.

## Results

### Patients’ characteristics

The baseline characteristics of the patients are shown in Table 3. The patients included 32 men and 16 women, with a median age of 76 years (IQR, 68–81 years) at surgery. The lesions were located in the esophagus in 15 patients (31%), pancreas in eight patients (17%), colon in seven patients (15%), stomach in six patients (13%), gall bladder in five patients (10%), duodenum in three patients (6%), liver in three patients (6%), and rectus in one patient (2%). The median duration (days) to start rehabilitation was 3 days (IQR, 2.0–5.25 days), while the median length of hospital stay was 21 days (IQR, 16.0–40.25 days).

**Table 3:** Patients’ characteristics

Sex	
Male (%)	32 (67%)
Female (%)	16 (33%)
Median: age (IQR)	76 (68–81)
Tumor	
Esophagus	15 (31%)
Pancreas	8 (17%)
Colon	7 (15%)
Stomach	6 (13%)
Gall bladder	5 (10%)
Duodenum	3 (6%)
Liver	3 (6%)
Rectum	1 (2%)
Median: rehabilitation treatment start date (IQR)	3 (2–5.25)
Median: length of stay (IQR)	21 (16–40.25)

*IQR* interquartile range

### Ambulation status

**Fig. 1:**
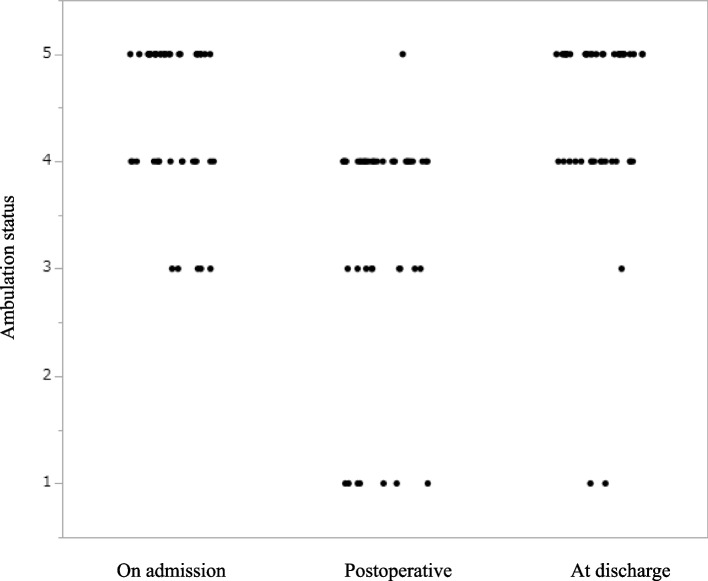
Perioperative course of mobilization. The evaluation was performed on admission, on the first day of rehabilitation after surgery, and at discharge. All patients reached or exceeded level 3 after surgery, except two patients who died of acute post operative events

Figure 1 shows the perioperative course of mobilization. Evaluations were performed on admission, on the first day of rehabilitation after surgery, and at discharge. On admission, 28, 15, and 5 patients had levels 5, 4, and 3. In most patients, the levels decreased on the first day of rehabilitation after surgery; however, progress was eventually achieved. All patients reached or exceeded level 3 after surgery, except for two patients who died of acute renal failure and bile leaks.

### Relationship between length of hospital stay, duration to start rehabilitation, and CCI scores

**Fig. 2:**
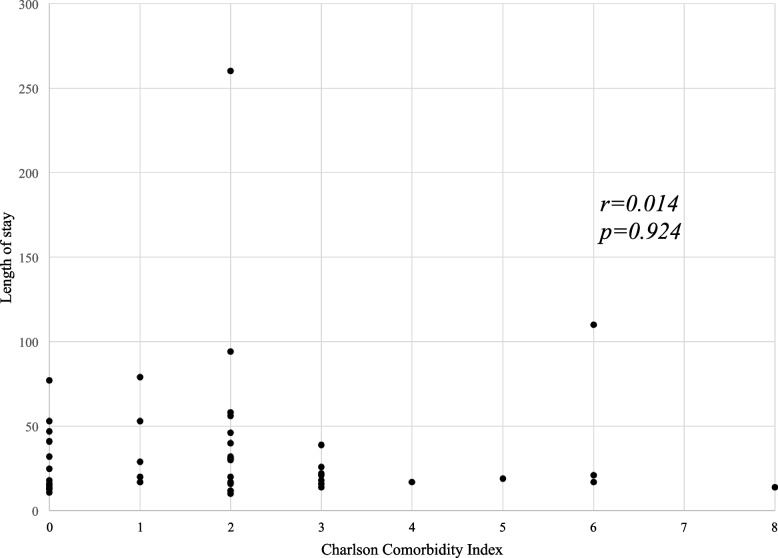
Relationship between the length of stay and the CCI scores

**Fig. 3:**
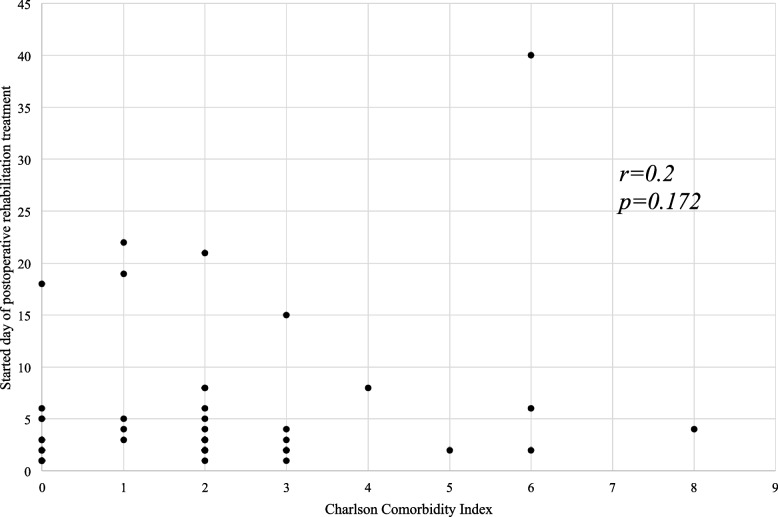
Relationship between the duration to start rehabilitation and the CCI scores

Figure 2 shows the relationship between length of stay and CCI scores. No significant correlation was observed between the two variables (*r* = 0.014, *p* = 0.924). Figure 3 shows the relationship between rehabilitation duration and CCI scores. Furthermore, no significant correlation was observed between the two variables (*r* = 0.2, *p* = 0.172).

### Complications not related to the CCI scores

**Fig. 4:**
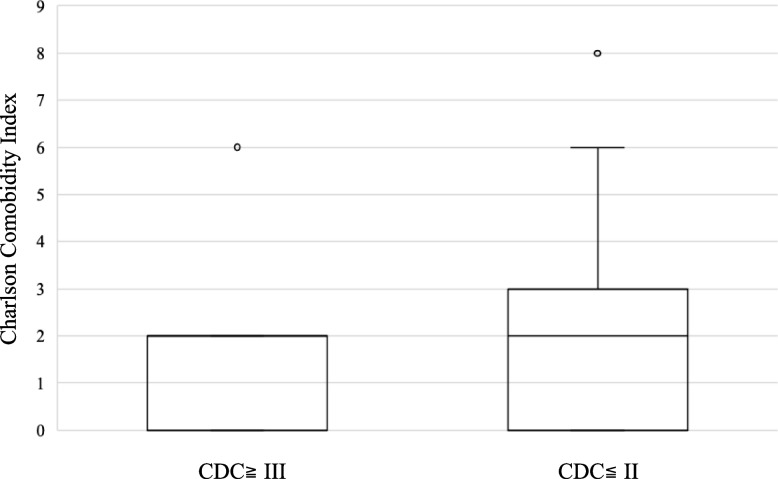
Charlson Comorbidity Index (CCI) scores in the patients with higher severity of surgical complications (CDC≧III) than in the patient with lower severity (CDC≦II)

**Fig. 5:**
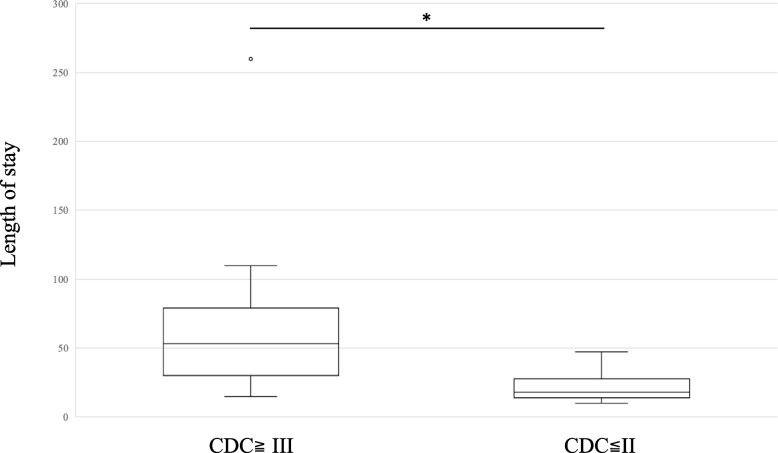
The length of stay in the patients with higher severity of surgical complications (CDC≧III) and patients with lower severity (CDC≦II)

**Fig. 6:**
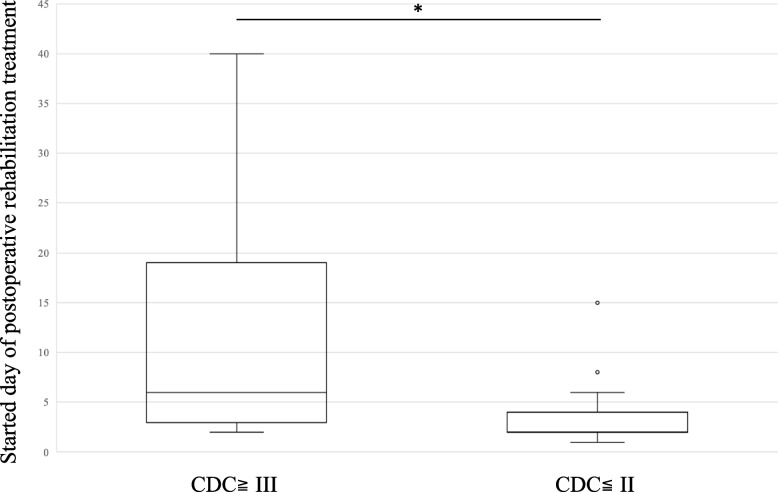
The duration to start rehabilitation in the patients with higher severity of surgical complications (CDC≧III) and patients with lower severity (CDC≦II)

Next, we determined the condition of the patients who could not reach or exceed Level 4 after rehabilitation. Sixteen patients met the inclusion criteria. Table 4 shows the conditions identified from the clinical records of the 16 patients. Interestingly, most conditions were related to surgery or “surgical complications” and were not included in the Charlson list. Therefore, we further assessed the clinical features of higher and lower severity complications in patients using the Clavien-Dindo classification. Figure 4 shows the CCI scores in the patients with higher severity of surgical complications (CDC≧III) and those with lower severity (CDC≦II). No significant differences were observed between the two groups. However, the length of stay and the duration to start rehabilitation were significantly longer in the patients with higher severity of surgical complications (CDC≧III) than in those with lower severity (CDC≦II, Figs. 5 and 6). We performed further statistical evaluation, including age and gender, but there was no statistical significance with length of hospital stay, duration to start rehabilitation, and the CCI scores.

**Table 4:** Conditions in patients who could not reach or exceed mobility level 4

Patient no	
1	Pain of chest drain
2	Bed rest indicated after resection with anastomosis
3	Heart failure
4	Pancreatic fistula
5	Bed rest indicated during transfusion
6	Tachycardia with hypertension
7	Delirium
8	Pain at the incision site
9	Delayed bleeding after surgery and reintubation
10	Vomiting and nausea
11	Abdominal abscess
12	Pain at the incision site (accompanied by sacrectomy)
13	Orthostatic hypotension
14	Bed rest indicated after resection with anastomosis
15	Spinocerebellar degeneration (mobility at level 3 prior to surgery)
16	Fever

## Discussion

Perioperative rehabilitation is of utmost importance in older patients with gastrointestinal cancer. This reduces the risk of complications, facilitates early recovery after surgery, and shortens the length of the hospital stay. Based on previous studies that reported that comorbidities worsen various conditions in cancer patients [14–16], we hypothesized that the CCI score predicts functional outcomes, including ambulation status, duration of rehabilitation post-surgery, and length of hospital stay. In terms of ambulation status, most patients reached or exceeded Level 3, suggesting the therapeutic effect of rehabilitation. We investigated the relationship between CCI scores and the duration of rehabilitation after surgery or the length of hospital stay but found no significant correlation.

Interestingly, we evaluated the clinical course of patients who did not reach or exceed Level 4 and identified conditions that were not in the CCI list (Table 1). Most of these conditions are associated with “surgical complications.” These conditions occurred in patients with low CCI scores.

Charlson et al. [21] designed CCI in 1987. They used data from an internal medicine inpatient service and analyzed the mortality rate at one year as a result of various comorbidities. A list of nineteen conditions was created. The index was validated in patients with breast cancer with a 10-year mortality rate. The CCI has been used and validated in predicting mortality risk in various conditions [28], while one study reported that comorbidity was not associated with functional status in older patients with cancer [29].

As a potential limitation of the CCI, Extermann et al. [28] suggested that the list ignores several comorbidities relevant to cancer treatment, such as hematopoietic disorders other than malignancies, polyneuropathy, and moderate renal dysfunction. This would be the key to interpreting our data; the CCI did not show a correlation with the duration (days) before the start of rehabilitation or the length of hospital stay. In patients who underwent surgery, “surgical complications” might be weighted heavier than the 19 conditions on the list in certain situations. We further investigated the clinical features of higher and lower severity complications in patients using the Clavien-Dindo classification. The patients with higher severity of surgical complications (CDC≧III) showed a longer length of stay and duration to start rehabilitation than those with lower severity (CDC≦II), suggesting the possible limitation of the CCI.

Our study has some limitations. This study was retrospective in nature, had a limited sample size, and was conducted at a single hospital. In addition, patients have different oncological backgrounds at different clinical stages. However, in our study, 19 conditions on the Charlson list were not sufficient to predict the functional outcomes of perioperative rehabilitation in patients with gastrointestinal cancer. Although the CCI is a useful tool for predicting oncological outcomes, treatment-related conditions may have a greater impact. In this study, we found that “surgical complications,” which are components of cancer treatment-related conditions, are also essential factors for assessing the therapeutic effect of rehabilitation. However, further large-scale studies in older cancer populations are required, particularly during the perioperative period. As society ages, the number of cancer patients with comorbidities will increase. To improve the quality of life of these elderly patients and their families, it is essential to identify factors that influence rehabilitation.

## Conclusions

Treatment-related conditions may significantly impact perioperative rehabilitation more than the original comorbidities. As well as original comorbidities, clinical events related to surgical complications should be assessed to determine the therapeutic effects of rehabilitation in patients with gastrointestinal cancer.

### Supplementary Information

**Additional file 1: Supplementary File 1.** Patient characteristics for surgery and perioperative rehabilitation.

## Data Availability

The datasets generated and/or analyzed during the current study are available from the corresponding author on reasonable request.

## References

[1] World health organization. Latest global cancer data: cancer burden rises to 19.3 million new cases and 10.0 million cancer deaths in 2020. https://www.iarc.who.int/faq/latest-global-cancer-data-2020-qa/. Accessed 13 Jul 2023

[2] Alves A, Panis Y, Mathieu P (2005). Postoperative mortality and morbidity in French patients undergoing colorectal surgery: results of a prospective multicenter study. Arch Surg.

[3] Richardson JD, Cocanour CS, Kern JA (2004). Perioperative risk assessment in elderly and high-risk patients. J Am Coll Surg.

[4] Duron JJ, Duron E, Dugue T (2011). Risk factors for mortality in major digestive surgery in the elderly: a multicenter prospective study. Ann Surg.

[5] Silver JK, Baima J (2013). Cancer prehabilitation: an opportunity to decrease treatment-related morbidity, increase cancer treatment options, and improve physical and psychological health outcomes. Am J Phys Med Rehabil.

[6] Silver JK, Baima J, Mayer RS (2013). Impairment-driven cancer rehabilitation: an essential component of quality care and survivorship. CA Cancer J Clin.

[7] Simić-Panić D, Bošković K, Milićević M (2018). The impact of comorbidity on rehabilitation outcome after ischemic stroke. Acta Clin Croat.

[8] Kabboord AD, van Eijk M, Fiocco M, van Balen R, Achterberg WP (2016). Assessment of comorbidity burden and its association with functional rehabilitation outcome after stroke or hip fracture: a systematic review and meta-analysis. J Am Med Dir Assoc.

[9] Radosavljevic K, Dragovic-Lukic G, Nikolic D (2020). Gender and musculoskeletal comorbidity impact on physical functioning in elderly after hip fracture: the role of rehabilitation. Healthcare (Basel).

[10] Scrutinio D, Giannuzzi P (2008). Comorbidity in patients undergoing coronary artery bypass graft surgery: impact on outcome and implications for cardiac rehabilitation. Eur J Cardiovasc Prev Rehabil.

[11] Schneider JC, Gerrard P, Goldstein R (2013). The impact of comorbidities and complications on burn injury inpatient rehabilitation outcomes. PM R.

[12] Horn SD, Smout RJ, DeJong G (2013). Association of various comorbidity measures with spinal cord injury rehabilitation outcomes. Arch Phys Med Rehabil..

[13] New PW, Earnest A, Scroggie GD (2017). A comparison of two comorbidity indices for predicting inpatient rehabilitation outcomes. Eur J Phys Rehabil Med.

[14] Kubo Y, Tanaka K, Yamasaki M (2021). Influences of the Charlson comorbidity index and nutrition status on prognosis after esophageal cancer surgery. Ann Surg Oncol.

[15] Liu X, Xue Z, Yu J (2020). Risk factors for postoperative infectious complications in elderly patients with gastric cancer. Cancer Manag Res.

[16] Dias-Santos D, Ferrone CR, Zheng H, Lillemoe KD, Fernández-Del CC (2015). The Charlson age comorbidity index predicts early mortality after surgery for pancreatic cancer. Surgery.

[17] Jensen BT, Laustsen S, Petersen AK (2013). Preoperative risk factors related to bladder cancer rehabilitation: a registry study. Eur J Clin Nutr.

[18] Chan CH, Maddison C, Reijnierse EM, Lim WK, Maier AB (2021). The association of comorbidity measures and mortality in geriatric rehabilitation inpatients by cancer status: RESORT. Support Care Cancer.

[19] Overview of the “Cancer Control Act”, Ministry of Health, Labour and Welfare, Japan. 2009. https://www.mhlw.go.jp/english/wp/wp-hw2/part2/p3_0026.pdf. Accessed 22 Sep 2023.

[20] Practical Guidance for Palliative Care Team, ver.2, Japanese Society of Palliative Medicine. https://www.jspm.ne.jp/files/english/guidance.pdf. Accessed 22 Sep 2023.

[21] Charlson ME, Pompei P, Ales KL, MacKenzie CR (1987). A new method of classifying prognostic comorbidity in longitudinal studies: development and validation. J Chronic Dis.

[22] Clavien PA, Sanabria JR, Strasberg SM (1992). Proposed classification of complications of surgery with examples of utility in cholecystectomy. Surgery.

[23] Dindo D, Demartines N, Clavien PA (2004). Classification of surgical complications: a new proposal with3evaluation in a cohort of 6336 patients and results of a survey. Ann Surg.

[24] Clavien PA, Barkun J, Oliveria ML (2009). The Clavien-Dindo classification of surgical complications: five-year experience. Ann Surg.

[25] Kim RY, Murphy TE, Doyle M (2019). Factors associated with discharge home among medical ICU patients in an early mobilization program. Crit Care Explor.

[26] Ishida Y, Shigematsu H, Tsukamoto S (2022). Case series of an impairment driven early ambulation program in cancer patients with cervical spine metastases after palliative spine surgery. Cancer Rehabil.

[27] Gerber LH, Valgo M, DeLisa JA, Gance BM (1998). Rehabilitatton for patients with cancer diagnoses. Rehabilitation Medicine: Principles and Practice.

[28] Extermann M (2000). Measuring comorbidity in older cancer patients. Eur J Cancer.

[29] Extermann M, Overcash J, Lyman GH, Parr J, Balducci L (1998). Comorbidity and functional status are independent in older cancer patients. J Clin Oncol.

